# Advancing small-animal molecular imaging through multifaceted innovation

**DOI:** 10.1117/1.JBO.31.5.054701

**Published:** 2026-05-01

**Authors:** Guosong Hong, Chongzhao Ran, Scott C. Davis, Kenneth M. Tichauer

**Affiliations:** a Stanford University, Department of Materials Science and Engineering, Stanford, California, United States; b Stanford University, Wu Tsai Neurosciences Institute, Stanford, California, United States; c Massachusetts General Hospital, Harvard Medical School, Athinoula A. Martinos Center for Biomedical Imaging, Charlestown, Massachusetts, United States; d Dartmouth College, Thayer School of Engineering, Hanover, New Hampshire, United States; e Lawson Research Institute, St. Josephs Health Care, London, Ontario, Canada; f Western University, Department of Medical Biophysics, London, Ontario, Canada

## Abstract

The editorial highlights articles in a JBO special section, as well as emerging trends in small-animal molecular imaging.

Molecular imaging in small animals has become an indispensable pillar of modern biomedical research, enabling the visualization of dynamic biological processes *in vivo* with high resolution, exceptional molecular specificity and sensitivity, and rapid turnaround with high throughput.^1^^,^^2^ Optical modalities, such as fluorescence, bioluminescence, and photoacoustic imaging, play a particularly important role in small-animal molecular imaging due to their high spatial and temporal resolution, non-ionizing nature, direct interface with molecular probes, and ease of implementation.^3^ A critical challenge in optical imaging of small animals arises from the inherently opaque nature of mammalian tissues, primarily due to strong scattering of photons, particularly in the visible range.^4^^–^^6^ Rooted in optical physics and light–tissue interactions, the three contributions in this JBO special section collectively highlight how dye-enabled optical clearing and shortwave infrared (SWIR) light can unlock new capabilities in small-animal imaging.^7^^,^^8^

The work of Sun et al. (featured on the issue cover) addresses a fundamental limitation of small-animal optical imaging, limited penetration depth, by engineering the optical properties of biological tissues themselves to achieve *in vivo* optical transparency using absorbing molecules.^7^ Building on prior discoveries that strongly absorbing dyes can modulate refractive index mismatches within tissue,^9^^–^^11^ the authors demonstrate that combining two absorbing molecules, tartrazine and 4-aminoantipyrine, provides a practical balance between clearing efficiency and biosafety. Their results show that optimized formulations enable rapid and effective tissue transparency in live animals while mitigating the toxicity associated with single-agent approaches. Importantly, this study highlights a recurring theme in molecular imaging: performance must be carefully balanced against physiological compatibility. While 4-aminoantipyrine offers superior penetration and clearing kinetics, its acute toxicity underscores the need for rational formulation design.

The authors envision that this optimized *in vivo* clearing cocktail can empower a range of optical imaging modalities, including light-sheet and confocal microscopy, in small animals. Beyond its immediate implications for deep-tissue imaging, this work reflects a broader conceptual shift in the field. Rather than solely designing improved probes or imaging systems, researchers are increasingly engineering the optical environment of the tissue itself.^12^^–^^14^ By modulating refractive index heterogeneity, such approaches reduce scattering at its physical origin, thereby enhancing imaging performance across a wide range of modalities, including photoacoustic microscopy, optical coherence tomography, and laser speckle contrast imaging.^15^^–^^19^ This emerging paradigm opens new avenues for noninvasive, longitudinal imaging in small animals.

Complementing these advances in engineering the optical properties of biological tissues, Patel et al.^20^ address a critical limitation in fluorescence imaging: the restricted dynamic range of SWIR detectors. SWIR imaging has emerged as a powerful modality due to reduced scattering and autofluorescence in the 1000-1700 nm window,^21^^,^^22^ yet its quantitative potential has been hindered by detector constraints. The authors introduce a high dynamic range (HDR) imaging framework specifically adapted to InGaAs cameras, incorporating detector-specific noise modeling and calibration strategies.

Their approach achieves a substantial extension in measurable dynamic range on the order of tens of decibels, enabling simultaneous quantification of signals spanning multiple orders of magnitude. This capability is particularly important in small-animal studies, where fluorophore distributions are inherently heterogeneous, with strong accumulation in organs such as the liver coexisting with weak signals in peripheral vasculature. By eliminating the need for exposure optimization and enabling robust radiometric calibration, HDR SWIR imaging represents a significant step toward fully quantitative optical molecular imaging.

Taken together, these two studies illustrate complementary strategies for overcoming optical limitations: one modifies the tissue, while the other enhances the detector and computational pipeline. Their convergence suggests a future in which both approaches are integrated by combining reduced scattering with expanded dynamic range to achieve unprecedented imaging performance.

The third contribution in this section, by Chue-Sang et al., addresses a different but equally important aspect of optical imaging: laser–tissue interaction and safety at infrared wavelengths.^8^ Understanding photonic safety thresholds is essential for the responsible deployment of optical technologies, particularly as higher energies and red-shifted wavelengths in the SWIR are increasingly used in both imaging and therapeutic contexts. Using a rabbit model, the authors quantify corneal damage thresholds for nanosecond laser pulses at 1645 nm, a spectral region with limited prior biological data. Their findings suggest that current safety standards may be overly permissive when evaluated using conventional averaging assumptions. Moreover, histological analysis reveals that tissue damage arises from a combination of photothermal and photomechanical mechanisms, rather than purely thermal effects.

This study emphasizes an often-underappreciated point: as imaging technologies push into new wavelength regimes such as SWIR, biological validation must keep pace with technological innovation. Accurate dosimetry and mechanistic understanding are critical not only for safety but also for interpreting imaging signals, particularly in modalities that rely on light-matter interactions at high intensities or short pulse durations.

From a broader perspective, the three papers in this special section collectively highlight several emerging trends in small-animal molecular imaging. First, advances in optical imaging increasingly depend on a deep understanding of optical physics to address complex biological questions. Second, techniques such as HDR SWIR imaging underscore the field’s transition from qualitative visualization toward reproducible, quantitative measurements. Third, innovations in contrast and clearing agents must carefully balance efficacy with safety, particularly for *in vivo* applications. Finally, the expanding use of SWIR wavelengths offers clear advantages in imaging performance but also introduces new challenges in instrumentation, calibration, and biological validation.

In conclusion, the contributions in this special section exemplify the dynamic and interdisciplinary nature of the field. By addressing fundamental limitations from multiple angles, including chemical, optical, and computational approaches, they collectively advance the frontier of molecular imaging in small animals. Looking ahead, the convergence of these directions points toward a future in which imaging strategies are holistically optimized across multiple dimensions, including tissue optical properties, probe design, detector performance, and computational reconstruction. Such integration will be essential for achieving the long-standing goal of noninvasive, high-resolution, and quantitatively accurate imaging deep within living organisms. These advances not only enhance current capabilities but also lay the foundation for future translational applications in diagnostics, therapeutics, and longitudinal biological studies.
